# The genetic landscape of inherited retinal dystrophies in Arabs

**DOI:** 10.1186/s12920-023-01518-7

**Published:** 2023-05-01

**Authors:** Lama Jaffal, Hawraa Joumaa, Jinane Noureldine, Malak Banjak, Mariam Ibrahim, Zamzam Mrad, Ali Salami, Said El Shamieh

**Affiliations:** 1 Department of Biological and Chemical Sciences, School of Arts and Sciences, Lebanese International University, Beirut, Lebanon; 2 Rammal Hassan Rammal Research Laboratory, PhyToxE Research Group, Faculty of Sciences, Lebanese University, Nabatieh, Lebanon; 3 Department of Mathematics, Faculty of Sciences, Lebanese University, Nabatieh, Lebanon; 4 Molecular Testing Laboratory, Department of Medical Laboratory Technology, Faculty of Health Sciences, Beirut Arab University, Beirut, Lebanon

**Keywords:** Inherited retinal dystrophies, Arabs, Country-based analysis, Genes, Mutations

## Abstract

Inherited retinal dystrophies (IRDs) are a major cause of vision loss. Altogether are highly heterogeneous genotypically and phenotypically, exhibiting substantial differences worldwide. To shed more light on these conditions, we investigated the genetic and phenotypic landscape of IRDs in the Arabs globally and per country.

We analyzed 1,621 affected individuals from 16 Arabic countries reported in 198 articles. At the phenotypic level, rod-cone dystrophy (RCD) and Usher syndrome were the most prevalent conditions among non-syndromic and syndromic IRDs. At the gene level, *TULP1, ABCA4, RP1, CRB1, MYO7A, RPE65, KCNV2*, and *IMPG2* were the most mutated genes. Interestingly, all except *CRB1* were highly prevalent because they harbored founder mutations, implying that consanguinity is a major determinant in Arab countries. Of note, ~ 93% of the investigated individuals carried homozygous mutations. The country analysis for the IRDs conditions and their associated genotypes revealed that whereas Leber Congenital Amaurosis, RCD, and USHER syndrome were widely distributed, bestrophinopathies and non-syndromic hearing loss were restricted to specific countries (till now).

This study could be a starting point for initiating suitable health policies towards IRDs in the Arab world. The high degree of homozygosity urges the need for genetic counsellors to provide personalized information and support the affected individuals.

**Supplementary Information:**

The online version contains supplementary material available at 10.1186/s12920-023-01518-7.

## Introduction

Inherited retinal dystrophies (IRDs) cover many rare retinal diseases affecting at least 1 in 2,000 individuals [1]. Altogether are known to be heterogeneous at the genetic and phenotypic levels [2]. IRDs often cause vision problems like night or color blindness, tunnel vision, and later total blindness,

all of which usually worsen with age [3]. Thus, they are a major cause of vision loss, impacting the patients’ and their families’ quality of life [4].

IRDs include at least 20 different conditions [1]. Affected individuals may show syndromic (extra-ocular symptoms) or non-syndromic symptoms if restricted to the eye, each having its specific age of onset, rate of progression, and causative gene(s) [3]. Retinitis pigmentosa (RP) [MIM: 268,000], also known as rod-cone dystrophy (RCD), is the most prevalent form [5]. Leber congenital amaurosis (LCA), cone or cone-rod dystrophies (CCRD), Stargardt disease (STGD), and best vitelliform macular dystrophy are other less frequent forms [2, 5]. On the other hand, syndromic IRDs are subcategorized, conforming to the syndrome’s type [5]. The most substantial common syndromic condition is Usher syndrome, a combination of hearing loss and RCD [6]. Other syndromic forms include Bardet-Biedl (BBS [MIM: 209,900]) and Alström syndrome (ALMS [MIM: 203,800]) [7].

In the era of precision medicine and gene therapy, genetic characterization of IRDs’ patients has become increasingly important [8]. More than 270 genes associated with IRDs have been reported (https://sph.uth.edu/retnet/). Every gene has its specific inheritance pattern and encodes a specific protein of different functions; structure, transmembrane, phototransduction, and visual cycle [9]. IRDs exhibits substantial genotypic and phenotypic differences across the world, analyzing this distribution in different populations is crucial for subsequent targeted therapeutic strategies [10]. Therefore, our goal was to identify the mutational spectrum and the IRDs distribution in the Arab countries globally, and per country, and to highlight the most prevalent mutations.

## Methods

### Ethics statement

Our systematic review analyzed public data made available in the published articles; thus, it does not require institutional review board approval. It was performed according to the preferred reporting items for systematic reviews and meta-analyses (PRISMA) [11].

### Literature search and study selection

We searched the PubMed and google scholar databases to retrieve human genetic studies investigating the IRDs in Arab countries (last accessed on April 30, 2022). Specifically, SES and LJ searched; Algeria, Bahrain, Comoros, Djibouti, Egypt, Iraq, Jordan, Kuwait, Lebanon, Libya, Mauritania, Morocco, Oman, Palestinian territories, Qatar, Saudi Arabia, Somalia, Sudan, Syria, Tunisia, United Arab Emirates, Yemen. The search terms were “inherited retinal disease OR inherited retinal degeneration OR retinal dystrophy” AND “ Algeria OR Bahrain OR Comoros OR Djibouti OR Egypt OR Iraq OR Jordan OR Kuwait OR Lebanon OR Libya OR Mauritania OR Morocco OR Oman OR Palestinian territories OR Qatar OR Saudi Arabia OR Somalia OR Sudan OR Syria OR Tunisia OR United Arab Emirates OR Yemen”. We also search using specific terms composed of ‘the name of every IRD and the Arab country’ to be as comprehensive as possible in retrieving the articles of interest.

All studies involving probands from any Arabic country mentioned before were included in the initial review. The inclusion criteria were as follows: human studies conducted on one or more of the populations defined above and with outcomes about IRD genotypes. Reference lists of the included studies were also screened to identify relevant articles. In contrast, studies that fulfilled the following criteria were excluded if: (1) focused on aspects other than the genetics of IRDs (animal models, treatment investigations). (2) written in a non-English language (because of the language barrier). (3) participants belong to non-Arabic-speaking Middle Eastern countries (i.e., Iran, Turkey), or the participants’ country is not specified. (4) No full text was available.

### Data collection

**Fig. 1:**
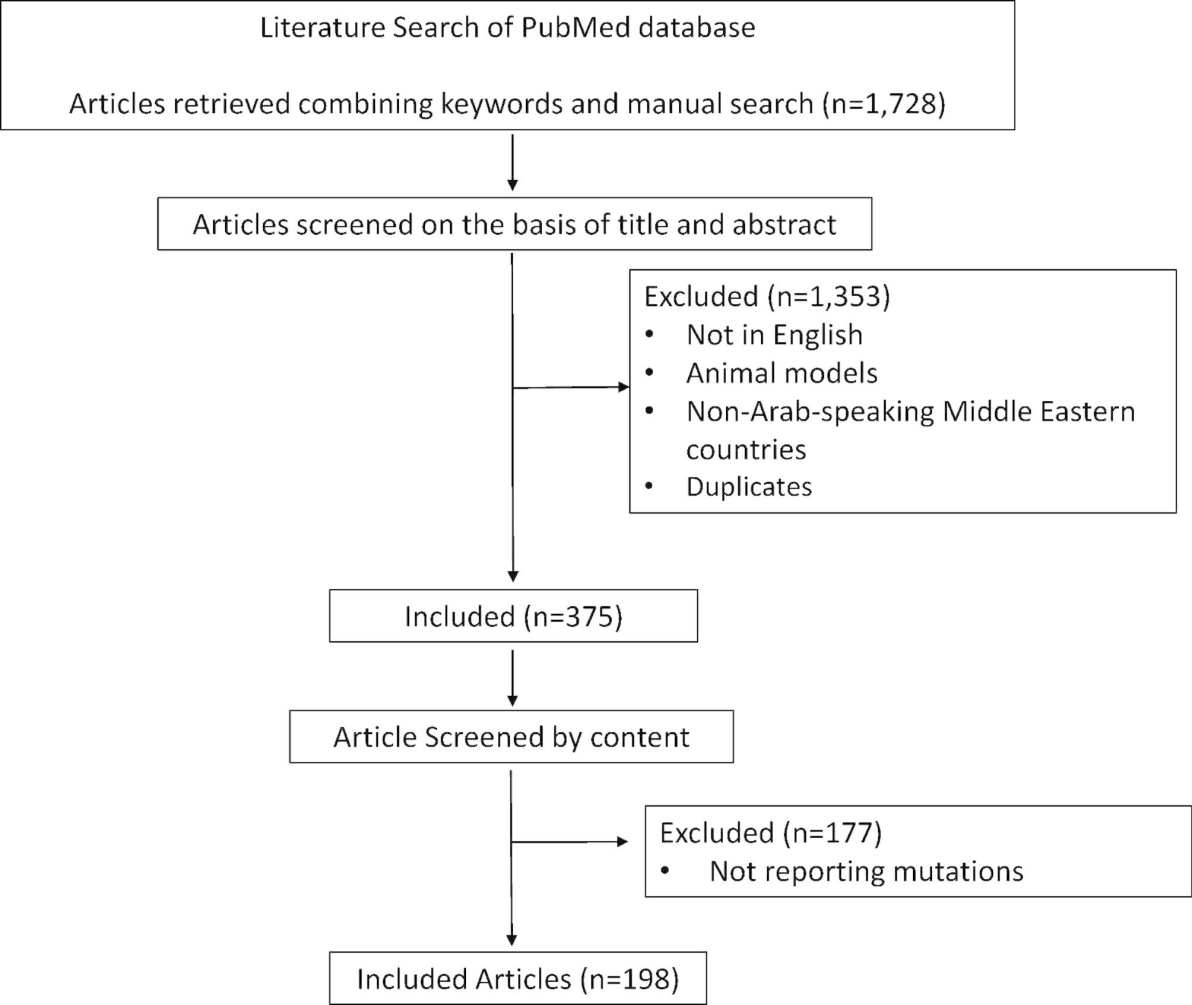
The flow chart for articles inclusion

Before compilation, data from the included studies and their supplementary data were extracted and summarized independently by two authors (MB, HJ, MI, JN, and ZM). All the previous filtering steps led to 198 articles published since 1996. The PRISMA flow diagram was summarized in Fig. 1.

The primary outcomes included documentation of the IRDs associated mutations in relation to ancestry. The associated IRD condition was also documented.

All analyses were conducted using SPSS software version 24 (SPSS, Inc, Chicago, Illinois). Categorical variables such as the type of IRD, the number of individuals were expressed as percentages. The histograms and pie charts were generated using Origin software (OriginPro, Version 2019b, OriginLab Corporation, Northampton, MA, USA).

## Results

**Fig. 2:**
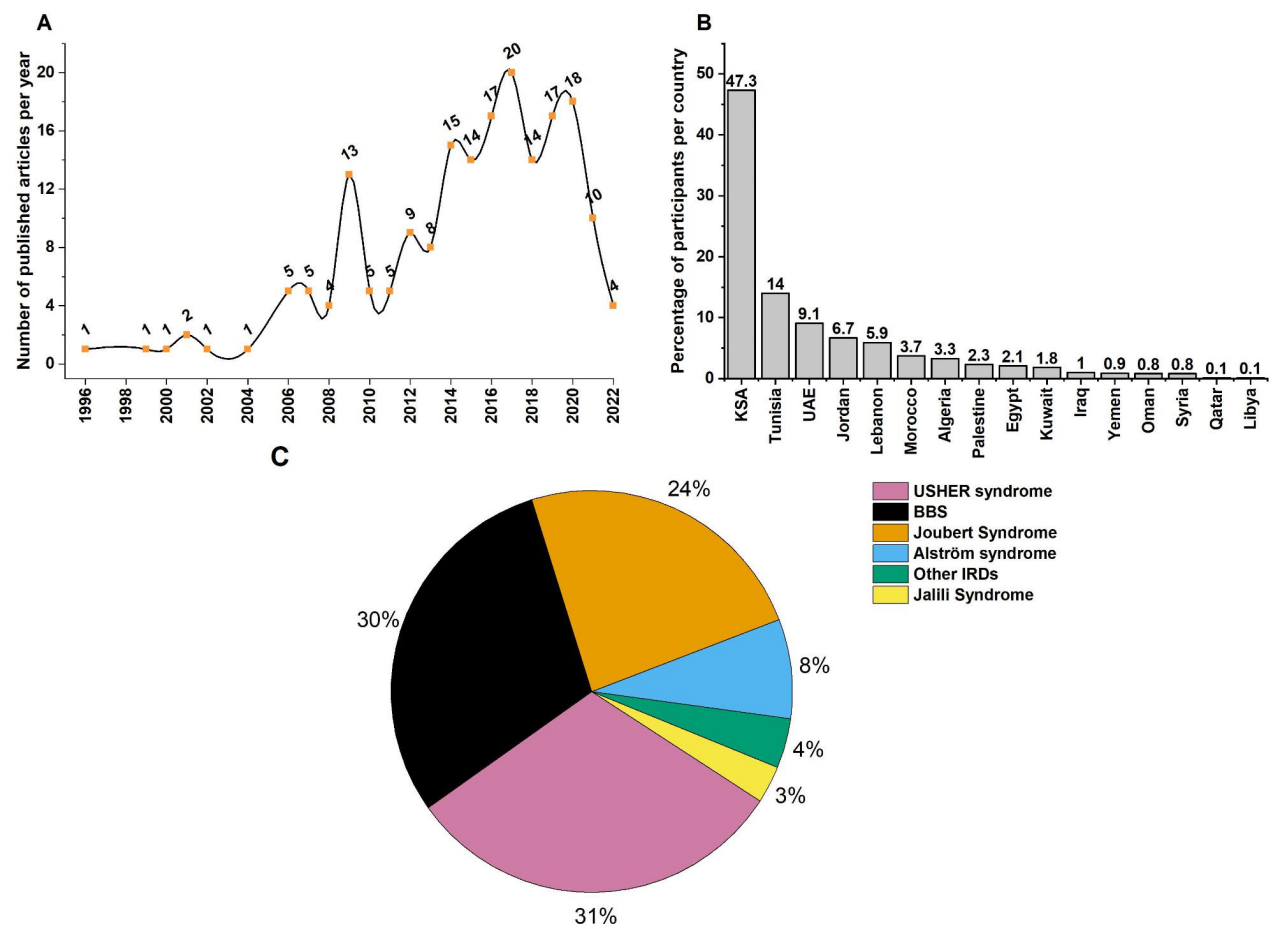
Articles, participants and distribution of syndromic inherited retinal diseases in the Arab world A- The number of articles investigating the genetics of inherited retinal diseases in the Arab-speaking countries until April 30, 2022. B. The distribution of the participants affected with inherited retinal diseases. C. Repartition of the different syndromic forms of inherited retinal diseases. BBS: Bardet-Biedl syndrome

Herein, we analyzed 198 articles that remained after the subsequent filtering steps (Fig. 1). The analysis of the publications output showed that only two of these articles were published before 2000, 39 additional followed during the first decade of the 2000s (Fig. 2A). The second decade revealed a four times increase in the publications’ output compared to the first decade (Fig. 2A). In total, 1,621 affected individuals from 16 different Arabic countries were reported around 47% of them were from Saudi Arabia (KSA), 14% from Tunisia, 9% from the United Arab Emirates, 7% from Jordan, 6% from Lebanon, 4% from Morocco, and 3% from Algeria (Fig. 2B). The remaining nine countries harbored 10% of the total number of participants. When analyzing the phenotypic repartition, we found that 83% of the participants had a non-syndromic form of IRDs. Among the syndromic forms, Usher syndrome (31%), BBS (30%) and Joubert Syndrome (24%) were the most prevalent conditions accounting for more than 85% of the published syndromic cases (Fig. 2C). Alström and Jalili syndromes were also found with a frequency of 8% and 3%, respectively (Fig. 2C). The remaining non-syndromic IRDs accounted for 4% of the total cases.

**Fig. 3:**
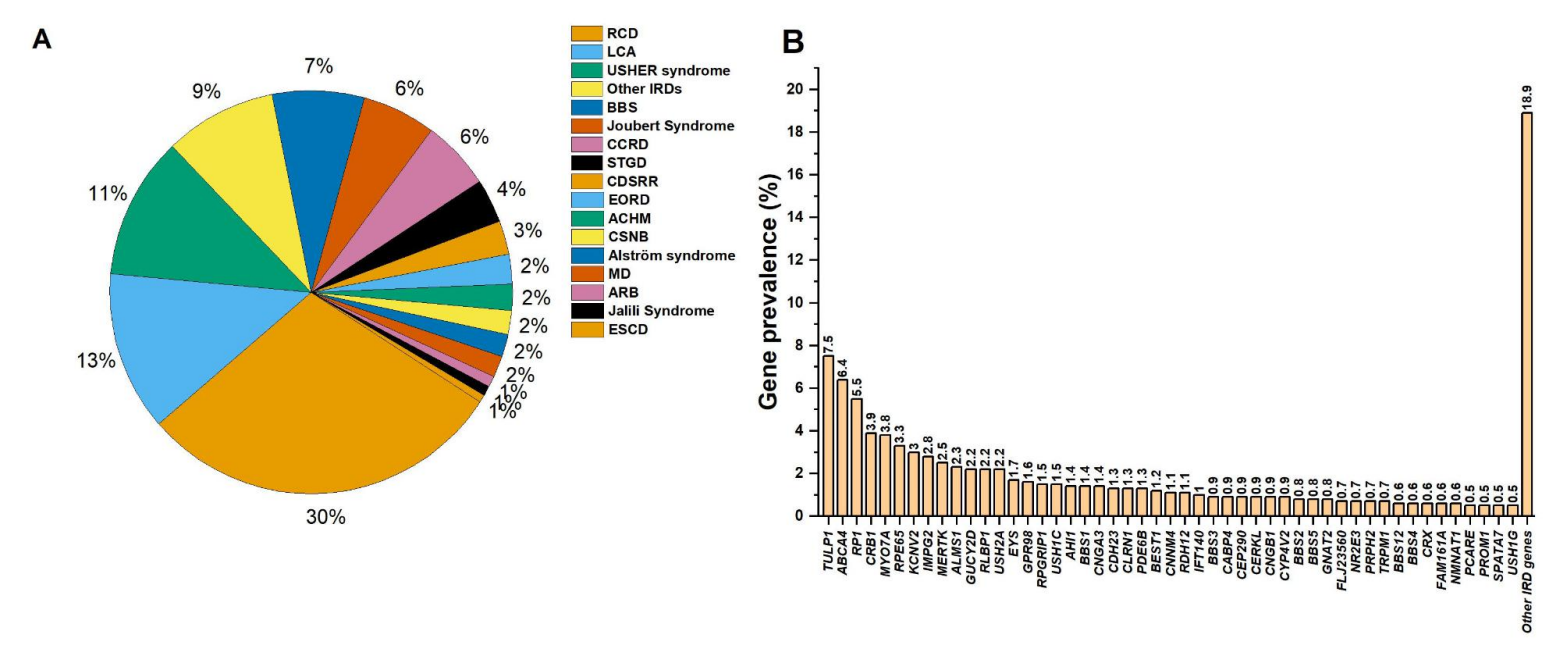
Prevalence of inherited retinal diseases and gene defects in Arabs The Pie charts shows the repartition of the inherited retinal diseases, whereas the histogram shows the prevalence of the gene defects RCD: rod-cone dystrophy, LCA: Leber congenital Amaurosis, IRDs: inherited retinal diseases, BBS: Bardet-Biedl Syndrome, CCRD: Cone or cone-rod dystrophy, CDSRR: cone dystrophy with Supernormal Rod Response, STGD: Stargardt disease, CSNB: congenital stationary night blindness, EORD: early onset of retinal dystrophy, MD: macular degeneration, ACHM: achromatopsia, ARB: autosomal recessive bestrophinopathy, ESCD: Enhanced S-cone dystrophy

Overall, at the phenotypic level, RCD was the most prevalent condition (30%) followed by Leber congenital amaurosis (13%), Usher (11%), BBS (7%), CCRD (6%) and Joubert Syndrome (6%) (Fig. 3A). Cone dystrophy with Supernormal Rod Response (CDSRR), Stargardt disease, congenital stationary night blindness, and early onset of retinal dystrophy were also detected among the Arabs but with lower frequencies (> 4%, Fig. 3A). Macular degeneration, achromatopsia, autosomal recessive bestrophinopathy (ARB), Jalili syndrome and Enhanced S-cone dystrophy had a frequency of 1-2% (Fig. 3A). The other forms of IRDs constituted altogether 9% of the total cases (Fig. 3B).

At the genotype level; *TULP1* (7.5%), *ABCA4* (6.4%), *RP1* (5.5%), *CRB1* (3.9%), *MYO7A* (3.8%), *RPE65* (3.3%), *KCNV2* (3%), *IMPG2* (2.8%), *MERTK* (2.5%), *ALMS1* (2.3%) were the most prevalent mutated genes (Fig. 3B). Variants in *USH2A* (2.2%) and *EYS* (1.7%) had a minor implication (Fig. 3B). All the remaining IRD genes had a prevalence of ≈ 19% of the total reported cases (Fig. 3B).

**Fig. 4:**
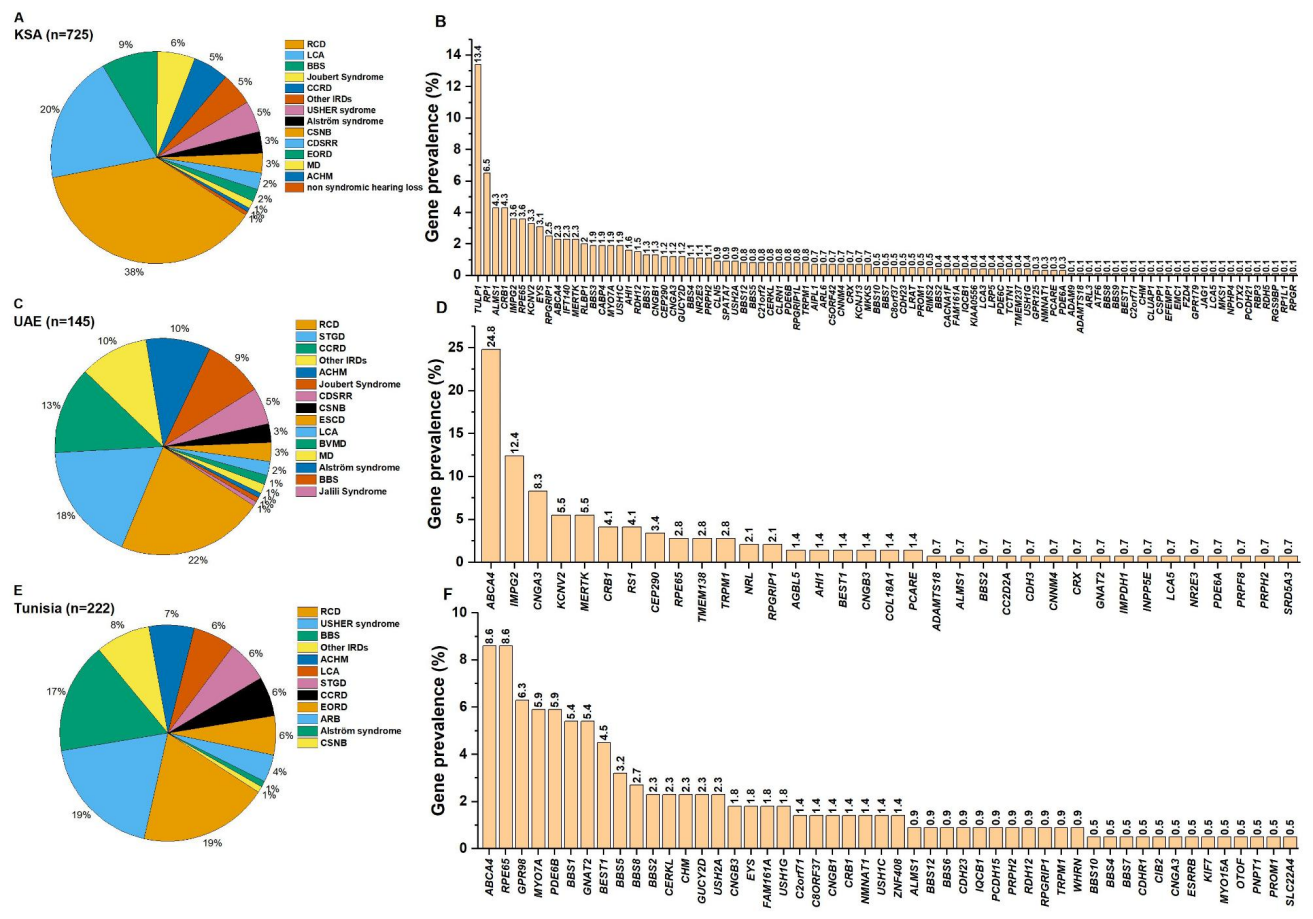
Prevalence of inherited retinal diseases and gene defects in specific Arab countries The Pie charts shows the repartition of the inherited retinal diseases, whereas the histogram shows the prevalence of the gene defects. KSA: Kingdom of Saudi Arabia, UAE: United Arab Emirates. n = number of participants

To go further in our analysis, we have performed a country-based analysis for the three countries with the highest number of participants (n): The Kingdom of Saudi Arabia (KSA); (n = 725, Fig. 4A), the United Arab Emirates (UAE); (n = 145, Fig. 4C), and Tunisia (n = 222, Fig. 4E). In KSA; at the phenotypic level, RCD (38%), LCA (20%), BBS (9%), and Joubert syndrome (6%) were the most prevalent IRDs responsible for more than two-thirds of the reported cases (Fig. 4A). At the DNA level, mutations in *TULP1* (13.4%), *RP1* (6.5%), *CRB1* and *ALMS1* (4.3%) were the three most frequent mutated genes (Fig. 4B).

In the UAE, RCD (22%), STGD (18%), CCRD (13%), and Achromatopsia (10%) were responsible for around two-thirds of the cases (Fig. 4C). On the other hand, *ABCA4* (24.8%), *IMPG2* (12.4%), and *CNGA1* (8.3%) were the most frequent mutated genes. In Tunisia, RCD (19%), Usher (19%), BBS (17%), and Achromatopsia (8%) were the most prevalent IRDs, making a total of 63% of the cases (Fig. 4D). At the genes’ level, *ABCA4* (9%), *RPE65 (*9%*), and GPR98* (6.3%) were the most mutated ones.

In addition to the countries mentioned above, we have also analyzed the IRDs’ distribution in Algeria, Lebanon, Morocco, Oman, and many others (Table 1). In Lebanon, a relatively high prevalence of Usher and BBS was observed (Table 1). We could notice that not all IRDs are widely distributed in the Arabic countries (at least till today). Whereas many conditions such as CCRD, Joubert syndrome, LCA, RCD, and USHER syndrome had a wide distribution, others were restricted. For example, till today ARB cases were only reported in Lebanon [12], and non-syndromic hearing loss was only reported in KSA [13] and Tunisia [14]. However, this conclusion relies should be taken with caution as it relies on a very limited sample size.

**Table 1:** The distribution of various types of inherited retinal diseases per country

	Phenotype																		
Origin	ACHM	Alström syndrome	ARB	BBS	BVMD	CCRD	CDSRR	CSNB	EORD	ESCD	Jalili Syndrome	Joubert Syndrome	LCA	MD	NSHL	Other IRDs	RCD	STGD	Usher syndrome
Algeria						1 (1.9)	5 (9.6)		5 (9.6)		1 (1.9)		10 (19.2)				2 (3.8)		28 (53.8)
Egypt						5 (14.7)	4 (11.8)					16 (47.1)	1 (2.9)			6 (17.6)			2 (5.9)
Iraq							3 (18.8)					5 (31.3)				1 (6.3)	3 (18.8)		4 (25.0)
Jordan						6 (6.3)					3 (3.2)	2 (2.1)	14 (14.7)			6 (6.3)	50 (52.6)		14 (14.7)
KSA	4 (0.6)	24 (3.3)		62 (8.6)		39 (5.4)	18 (2.5)	22 (3.0)	14 (1.9)		2 (0.3)	42 (5.8)	142 (19.6)	9 (1.2)	4 (0.6)	32 (4.4)	274 (37.8)	2 (0.3)	35 (4.8)
Kuwait														16 (55.2)			13 (44.8)		
Lebanon			6 (6.4)	13 (13.8)				2 (2.1)	2 (2.1)		3 (3.2)	1 (1.1)	3 (3.2)			16 (17.0)	5 (5.3)		43 (45.7)
Libya												1 (100)							
Morocco							4 (6.8)			3 (5.1)		3 (5.1)				24 (40.7)	20 (33.9)		5 (8.5)
Oman		1 (7.7)		4 (30.8)								5 (38.5)				3 (23.1)			
Palestine						2 (5.4)				3 (8.1)	2 (5.4)	1 (2.7)	8 (21.6)			8 (21.6)	3 (8.1)	10 (27.0)	
Qatar						2 (100)													
Syria						2 (16.7)			1 (8.3)			1 (8.3)	3 (25.0)				5 (41.7)		
Tunis	15 (6.8)	2 (0.9)	9 (4.1)	37 (16.7)	1 (0.5)	13 (5.9)		2 (0.9)	13 (5.9)				14 (6.3)		1 (0.5)	16 (7.2)	43 (19.4)	14 (6.3)	42 (18.9)
UAE	14 (9.7)	1 (0.7)		1 (0.7)	2 (1.4)	19 (13.1)	8 (5.5)	4 (2.8)		4 (2.8)	1 (0.7)	13 (9)	3 (2.1)	2 (1.4)		15 (10.3)	32 (22.1)	26 (17.9)	
Yemen		1 (6.7)										1 (6.7)	4 (26.7)				2 (13.3)		7 (46.7)

Data are shown as numbers (n) and percentages (%)

ACHM: achromatopsia, arb: Autosomal recessive bestrophinopathy, BBS: Bardet-Biedl Syndrome, BVMD: Best vitelliform macular dystrophy, CCRD: Cone or cone-rod dystrophy, CDSRR: cone dystrophy with supernormal rod response, CSNB: congenital stationery night blindness, EORD: Early onset of retinal dystrophy, LCA: Leber congenital amaurosis, RCD: Rod-cone dystrophy, ESCD: Enhance S cone dystrophy, JS: Joubert syndrome, LCA: Leber congenital amaurosis, MD: macular degeneration, NSHL: non-syndromic hearing loss, IRDs: inherited retinal dystrophies, STGD: Stargardt disease. RCD: Rod-cone dystrophy

The most prevalent mutations in Arabic-speaking countries were shown in Table 2. The nonsense mutation c.901 C > T; p.(Gln301*) in *TULP1* remains the most prevalent mutation in the Arab world, with more than 100 affected individuals [15]. Three phenotypes were associated with this mutation: CCRD, LCA, and RCD (Table 2). Two *RP1* mutations; c.606 C > A; p.(Asp202Glu) and c.3428del; p.(Asn1143Ilefs*25) *w*ere also prevalent and associated with RCD and macular dystrophy in KSA and Kuwait (Table 2). The nonsense mutation c.427G > T; p.(Glu143*) in *KCNV2* was prevalent UAE and KSA and was associated with CCRD and cone dystrophy with supernormal rod response. The missense mutation c.5882G > A; p.(Gly1961Glu) in *ABCA4* was the most prevalent  in UAE individuals and was associated with CCRD and STGD (Table 2). Another missense mutation; c.332T > C; p.(Ile111Thr) in *CYP4V2* showed a high prevalence in Lebanese individuals with Bietti crystalline dystrophy. Almost all the reported mutations except c.470 + 1G > A; p.(=) in *MYO7A* and c.5668G > T; p.(Gly1890*) in *CEP290* were associated with various IRDs, implying a remarkable degree of phenotypic heterogeneity. Most of these genes showed a high degree of phenotypic variability and their mutations were restricted to specific for countries. The ‘exceptions’ were; c.427G > T; p.(Glu143*) in *KCNV2*,* c.5882G > A; p.(Gly1961Glu)* in *ABCA4* and c.5668G > T; p.(Gly1890*) in *CEP290*.

**Table 2:** The most prevalent IRDs’ mutations in the Arab World

Gene	Mutation	Country	N	Inherited Retinal Disease
*TULP1*	c.901 C > T; p.(Gln301*)	KSA	97	CCRD
				LCA
				RCD
		Arabian Peninsula	10	RCD
*RP1*	c.606 C > A; p.(Asp202Glu)	Kuwait	29	MD
				RCD
		KSA	5	RCD
*KCNV2*	c.427G > T; p.(Glu143*)	KSA	23	CDSRR
				CCRD
				RD
		UAE	7	CDSRR
*ABCA4*	c.5882G > A; p.(Gly1961Glu)	Mainly UAE	30	CCRD
				STGD
*IFT140*	c.1990G > A; p.(Glu664Lys)	KSA	16	LCA
				EORD
				RD
				IFT140-related retinal‐ renal ciliopathy
*RP1*	c.3428del; p.(Asn1143Ilefs*25)	KSA	16	RCD
*IMPG2*	c.189dup; p.(Gln64Thrfs*9)	UAE	12	CCRD
				RCD
*MERTK*	c.2214del; p.(Cys738Trpfs*32)	KSA	12	RCD
*MYO7A*	c.470 + 1G > A; p.(=)	KSA	6	USHER
		Algeria	1	
		Tunisia	5	
*CABP4*	c.81_82insA; p.(Pro28Thrfs*4)	KSA	12	CSNB
				LCA
*RPE65*	c.271 C > T; p.(Arg91Trp)	KSA	9	LCA
		Tunisia	2	RCD
*CYP4V2*	c.332T > C; p.(Ile111Thr)	Lebanon	11	BCD
*RPE65*	c.271 C > T; p.(Arg91Trp)	Tunisia	11	EORD
*CEP290*	c.5668G > T; p.(Gly1890*)	KSA	5	
		UAE	5	JS
		Oman	?	

N: Number of affected individuals. KSA: Kingdom of Saudi Arabia, CCRD: Cone or cone-rod dystrophy, LCA: Leber congenital amaurosis, RCD: Rod-cone dystrophy, N.A: Not applicable, MD: macular degeneration, CDSRR: cone dystrophy with supernormal rod response, UAE: United Arab Emirates, STGD: Stargardt disease, EORD: Early onset of retinal dystrophy, RD: Retinal dystrophy, BCD: Bietti crystalline dystrophy, JS: Joubert Syndrome

## Discussion

We have previously shown that RCD distribution has substantial differences in the Arab countries [15, 16]. To go further, we have now expanded our analysis by investigating the repartition of all IRDs and their genetic mutations in the Arab countries. Herein, we showed that the research output of Arab countries focusing on IRDs has significantly increased since 2012; this is reflected by the increase in the number of articles using next-generation sequencing. To some extent, this part of the world has ‘benefited’ from the NGS revolution. However, a lot remains to be done, especially in countries in the middle east and north Africa where the number of genetically characterized individuals is low.

One common finding in all our analyses is that RCD and Usher syndrome were the most prevalent non-syndromic and syndromic IRDs, respectively (Figs. 2 and 3). In the global analysis, RCD, LCA, and CCRD were the most prevalent non-syndromic forms (Fig. 2). On the other hand, USHER, BBS, and Joubert syndromes were the most prevalent syndromic conditions (Fig. 2). The country analysis showed a similar trend with the global one but with some exceptions (Fig. 4). For instance, in UAE, STGD disease was more prevalent than LCA (Fig. 4C). In Tunisia, STGD was the third most prevalent non-syndromic IRD condition preceding CCRD (Fig. 4E).

At the gene level, *TULP1*, *ABCA4*, *RP1, CRB1, MYO7A, RPE65*, *KCNV2*, and IMPG2 were the most mutated genes globally (Fig. 3). Interestingly, all the mentioned genes except *CRB1* were the most prevalent because they showed harbored founder mutations (Table 2). This finding shows that consanguinity is a major determinant of gene prevalence in Arab countries; this is highlighted by the high frequency of homozygous mutations in the affected individuals covered in the current study (~ 93%). In our analysis, *USH2A* was not a major IRD gene despite that RCD and Usher were two of the most prevalent IRD conditions globally and per country. We have previously shown that *USH2A* was a minor gene in Arab RCD patients [15]. A recent study focusing on Asiatic IRD individuals (from Taiwan) showed that *USH2A* mutations were prevalent in sporadic IRD patients, but not in those with a family history [17]. One explanation for the low prevalence of *USH2A* mutations in Arab countries might be the high consanguinity and the founder effects resulting that only a minority of Arabs with IRD are sporadic. However, this remains a speculation with no supporting data.

The country-based analysis showed that in KSA: *TULP1*, *RP1*, and *ALMS1* were the most mutated genes. In contrast, *ABCA4* mutations were the most common in UAE, followed by *IMPG2* and *CNG3* mutations. In Tunisia, *ABCA4* was also the most mutated, followed by *RPE65* and *GPR98*. Although *ABCA4* is the most mutated in UAE and Tunisia, it showed a different mutational pattern. The majority of UAE individuals carried the frequent mutation: p.(Gly1961Glu), whereas the Tunisian individuals were more heterogeneous genetically and carrying various mutations such as p.(Gly1087Lys), p.(Trp782*), c.?_-463_5714+?dup?_6148_6479_+del; p.(Arg681*). *ABCA4* mutations are known to be associated with STGD [18]. *ABCA4* harbors numerous founder alleles with a specific geographical territory [19] and exhibits a significant variation in disease-causing alleles across different ethnic backgrounds [19]. The most common *ABCA4* disease-causing mutation, p.(Gly1961Glu), seems to have appeared first in Eastern Africa, where it is present in frequencies of about 8–10% in Somalia, Kenya, and Ethiopia [19]. Due to population migration, the p.(Gly1961Glu) has dispersed throughout the world and in different Arabic countries such as UAE [23], Jordan [24]. In Tunisia, the variant p.(Gly1961Glu) is yet absent while the p.(Gly1087Lys) in *ABCA4* is the most common mutation.

Countries such as Lebanon and Jordan are increasingly providing genetic diagnosis for their IRD individuals. However, the data gathered from these two countries cannot provide a reliable source (< 100 IRD individuals in each country) for genotype-phenotype association analysis.

In general, Arabs are genetically diverse [20]. Different countries in the gulf region have a relatively homogeneous population [20]. While countries like Lebanon and Tunisia have admixture populations because of their long history of trading across the Mediterranean sea [20]. Familial Mediterranean Fever, for example, is prevalent in Lebanon, Jordan, and among Palestinians while nonexistent in the Arabian Peninsula [20]. In addition, although the consanguinity rates are generally high in all Arab countries, they differ across those countries, ranging from 25% in Lebanon [21], up to 60% in KSA [22], and 90% in some Bedouin tribes. Going into the same direction, IRD phenotypes and genotypes showed similar specificity across countries.

This is the first study characterizing the genetic and phenotypic aspects of IRDs in the Arab countries. However, several limitations need to be mentioned: (1) the inclusion criterion ‘Arabic speaking countries’ was used instead of the ethnic and racial categories because no genetic information on the ancestry is available. (2) A significant proportion (47%) of the participants were from KSA. This is not surprising because other Arab countries do not have comparable resources and personnel. (3) Most IRD patients lack published phenotypic data; thus, we could not check the adequacy of genotype-phenotype associations. (4) The investigators removed the duplicate individuals between studies by comparing their IDs, country of origin, IRD condition, and associated mutations; despite this, many duplicates might have been missed if the authors used different ID across different studies.

Our work is the first milestone in organizing the existing genetic data about IRDs in Arabs in a population-specific manner and highlighting the distribution of causative genes. The high degree of homozygosity urges the need for genetic counsellors to provide personalized information and support the affected individuals. If used properly, this catalog might be a starting point for intiating suitable health policies towards these ‘common’ yet considered rare diseases. Suitable health policies, could benefit the therapeutic strategies. More studies in the Arab world especially in its understudied countries are needed before drawing definitive conclusions.

## Electronic supplementary material

Below is the link to the electronic supplementary material.

Supplementary Material 1

Supplementary Material 2

## Data Availability

All data generated or analysed during this study are included in this published article as supplementary information.
